# LecT-Hepa facilitates estimating treatment outcome during interferon therapy in chronic hepatitis C patients

**DOI:** 10.1186/1559-0275-11-44

**Published:** 2014-12-11

**Authors:** Xia Zou, Xiumei Chi, Yu Pan, Dongning Du, Haibo Sun, Atsushi Matsuda, Wei Li, Atsushi Kuno, Xinxin Zhang, Hisashi Narimatsu, Junqi Niu, Yan Zhang

**Affiliations:** Ministry of Education Key Laboratory of Systems Biomedicine, Shanghai Center for Systems Biomedicine, Shanghai Jiao Tong University, 800 Dong Chuan Road, Minhang Shanghai, 200240 China; Department of Hepatology, First Hospital, Jilin University, Changchun, 130021 China; Research Center for Medical Glycoscience (RCMG), National Institute of Advanced Industrial Science and Technology (AIST), 1-1-1 Umezono, Tsukuba, Ibaraki, 305-8568 Japan; SCSB (China) - AIST (Japan) Joint Medical Glycomics Laboratory, 800 Dong Chuan Road, Minhang Shanghai, 200240 China; Department of Infectious Diseases, Ruijin Hospital, Shanghai Jiao Tong University School of Medicine, 197, Ruijin Er Road, Shanghai, 200025 China; Department of Surgery, Ruijin Hospital, Shanghai Jiao Tong University School of Medicine, 197, Ruijin Er Road, Shanghai, 200025 China

**Keywords:** Glycoprotein, LecT-Hepa, Non-invasive, Treatment outcome, CHC

## Abstract

**Background:**

A combination treatment of interferon and ribavirin is the standard and the commonly used treatment for chronic hepatitis C (CHC). Developing noninvasive tests like serum indicators that can predict treatment outcome at an early stage of therapy is beneficial for individualized treatment and management of CHC. A glyco-indicator based on the glyco-alteration of serum α1-acid glycoprotein, LecT-Hepa, was discovered by glycomics technologies as a robust indicator of liver fibrosis. Here, we investigated the clinical utility of LecT-Hepa for evaluation of treatment outcome.

**Results:**

Firstly, ninety-seven patients with CHC were used for comparison of LecT-Hepa in serum and plasma. We found no significant difference in the concentrations of LecT-Hepa in serum and plasma. And then, 213 serum specimens from 45 patients who received 48 weeks of treatment with interferon and ribavirin were followed up for 96 weeks, and were used for evaluation of the role of LecT-Hepa. We found that LecT-Hepa might reflect the change in fibrosis regression during the treatment process. Moreover, the change of LecT-Hepa at the first 12 weeks of treatment could already predict the antiviral treatment response, which was more superior to FIB-4 index and aspartate aminotransferase-to-platelet ratio index (APRI) in this study.

**Conclusions:**

These results provide a new perspective that serum glycoprotein could be used as a joint diagnosis indicator for estimation treatment outcome of viral hepatitis at earlier stage of therapy.

**Electronic supplementary material:**

The online version of this article (doi:10.1186/1559-0275-11-44) contains supplementary material, which is available to authorized users.

## Introduction

Chronic hepatitis C virus (HCV) infection is a highly prevalent public health concern and one of the leading causes of cirrhosis, hepatocellular carcinoma, and liver failure [1]. An estimated 150 million people worldwide are chronically infected with HCV, and >350,000 people die from hepatitis-C-related liver diseases every year [2]. The standard treatment widely used for chronic hepatitis C (CHC) is a combination of peginterferon and ribavirin [3, 4]. The indication of successful therapy is the attainment of sustained virological response (SVR), which is defined as undetectable serum HCV RNA 24 weeks after treatment cessation [5]. With the current standard treatment, patients with chronic HCV infection show an SVR rate of ~55% [6, 7]. This means that there is a large population of patients with treatment outcomes of no response, virological breakthrough, or relapse. Early prediction of the outcome during or after treatment is expected to provide additional information for individualizing treatment, and thus improves the cure rates for patients with chronic HCV infection.

One of the most important histological outcomes of interferon (IFN) therapy is the change in degree of fibrosis. Many studies have clearly shown that IFN therapy results in significant regression of fibrosis in patients who attain SVR [8–10]. Thus, continuous monitoring of the degree of liver fibrosis should be beneficial for early estimation of the therapeutic efficacy and long-term follow-up of patients, which provides clues for the prognosis and management of CHC. It is evident that liver biopsy is considered as the gold standard for fibrosis staging [11]. This procedure has several disadvantages including invasiveness, potential complications, and sampling errors, which often limit its application, for example, frequent monitoring of the degree of fibrosis [12–14]. The development of noninvasive methods to complement liver biopsy is urgently needed. From this point of view, a variety of noninvasive methods has been developed, including physical techniques such as FibroScan [15] and serological tests such as FibroTest, Hepascore, enhanced liver fibrosis (ELF) index, platelets, APRI, and FIB-4 index [16–19]. FibroScan is recognized as a superior test for evaluation of fibrosis compared with biochemical markers [20]. It is restricted by the cost and the operator’s experience and patient’s body mass index (BMI) [21]. Many serological methods are also moderately useful for identifying significant fibrosis or cirrhosis in patients with chronic HCV infection. However, there are few serological tests reported to meet the above medical need.

Our previous study using glycomics technologies have developed and revealed a new fibrosis test named LecT-Hepa, which measures a glycobiomarker serum α1-acid glycoprotein (AGP) with fibrosis-related glyco-alterations performed well in estimating liver fibrosis [22]. It is correlated well with the fibrosis stage determined by liver biopsy [22–24] and FibroScan [25], either in a single-center [22, 23] or a multicenter study [24]. In the present study, continuous use of LecT-Hepa as an indicator of liver fibrosis during 48 weeks therapy with IFN and ribavirin led us to predict the outcome within the treatment period. We found that the change of LecT-Hepa just at the first 12 weeks of therapy could already distinguish CHC patients’ attainment of SVR.

## Results

### Evaluation of the level of LecT-Hepa in serum and plasma specimens

**Figure 1:**
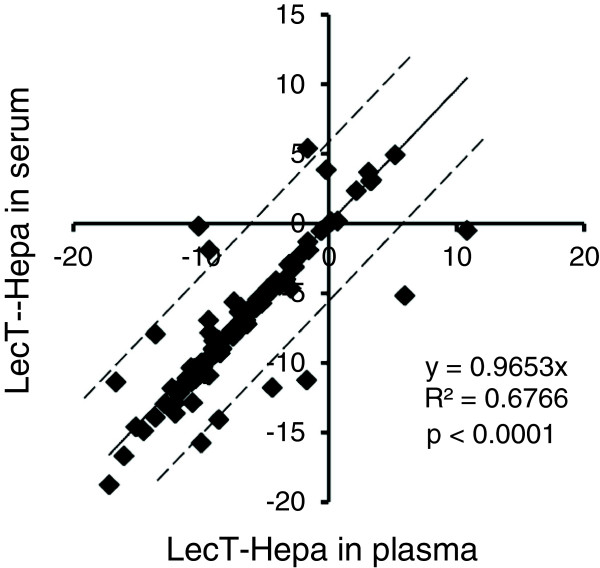
**Correlation of concentrations of LecT-Hepa in serum and plasma specimens prepared simultaneously from the same individuals.** The linear regression analysis was performed in 97 patients with confirmed CHC. The best-fit linear comparison with its correlation coefficient was calculated in Excel 2007 (Microsoft). The dotted line shows the 95% confidence intervals of the correlation.

LecT-Hepa has been shown as a reliable method for the evaluation of liver fibrosis [22, 24, 25]. However, previous studies were all conducted using serum specimens. To broaden the clinical application of LecT-Hepa, we compared the concentration of LecT-Hepa in serum and plasma prepared simultaneously from the same individuals. A total of 97 patients with confirmed CHC were included for this comparison (Table 1). As shown in Figure 1, we observed a significant linear correlation between the level of LecT-Hepa in serum and that in plasma (R^2^ = 0.6766, p < 0.0001), with most of the patients (89 of 97) within the 95% confidence intervals of the correlation. In addition, according to the best linear curve with its correlation coefficient (y = 0.9653x), the concentrations of LecT-Hepa in serum and plasma were almost the same. These data suggest that the serum and plasma specimens could both be used for clinical detection of LecT-Hepa.

**Table 1:** **Clinical characteristics of the 142 CHC patients in this study**

	CHC patients with serum and plasma specimens (n = 97)	CHC patients treated with IFN and achieved RVR (n = 45)
Age (year)	52.30 ± 8.20	52.64 ± 7.49
Gender (male/female)	55/42	30/15
TBIL (μmol/L)	16.90 ± 7.40	/
DBIL (μmol/L)	7.47 ± 3.14	/
ALP (U/L)	88.41 ± 31.01	/
GGT (U/L)	79.79 ± 91.21	/
ALT (U/L)	57.88 ± 50.87	119.71 ± 110.95
AST (U/L)	45.50 ± 32.49	87.70 ± 82.70
PLT (×10^9^/L)	189.07 ± 66.50	166.60 ± 83.20
FibroScan	9.44 ± 10.22	15.16 ± 7.63
MAL/DSA	Serum: 10.01 ± 1.94	9.29 ± 2.39
	Plasma: 10.35 ± 2.30	
AOL/DSA	Serum: 3.02 ± 3.43	6.34 ± 7.33
	Plasma: 3.57 ± 4.78

### Baseline characteristics of 45 patients achieved rapid virological response (RVR)

A total of 45 CHC patients who had achieved RVR during IFN therapy and undergone 2 years of follow-up were used for the evaluation of the role of LecT-Hepa during hepatitis C treatment and follow-up. The mean age of the 45 patients was 52.64 ± 7.49 years, and 30 (66.7%) of them were men (Table 1). To investigate the relation between the level of LecT-Hepa and fibrosis, these patients were divided into three groups based on the degrees of severity of liver fibrosis assessed by FibroScan. According to the study by Berzigotti et al. [26], 18 (40.0%) patients with FibroScan value <12 kPa and 13 (28.9%) with FibroScan value ≥18 kPa were considered as non-cirrhosis (non-LC) and cirrhosis (LC), respectively. Other patients with FibroScan values of 12–18 kPa were indeterminate. We also assessed the degree of fibrosis using color Doppler ultrasound. The results were highly consistent with the assessment by FibroScan (Table 2). In addition, the baseline characteristics of these patients are also summarized in Table 2. For the routine clinical indicators, platelet count (PLT) showed the most significant differences (p = 0.004 for non-LC *vs.* indeterminate, and p = 0.001 for non-LC *vs.* LC respectively, Student’s *t* test), while other indicators such as sex, alanine aminotransferase (ALT), and BMI did not differ significantly among these three groups. However, for the new indicator, both MAL/DSA and AOL/DSA always showed significant differences among the three groups. In addition, the univariate analysis revealed that the most significant differences were found between the non-LC and LC groups, whereas the indeterminate and LC groups showed no difference. We observed a significant decrease in the level of MAL/DSA (p = 2.68E-06 *vs.* non-LC) and an increased level of AOL/DSA (p = 0.004 *vs.* non-LC) in the LC group. These results suggest that the new indicator LecT-Hepa may be superior to the routine clinical indicators for the evaluation of fibrosis in this cohort.

**Table 2:** **Baseline characteristics of the 45 HCV patients in three different groups**

	Non-LC (n = 18)	Indeterminate (n = 14)	LC (n = 13)	Significance		
				Non-LC ***vs***indeterminate	Indeterminate ***vs***LC	Non-LC ***vs***LC
Age (year)	49.28 ± 5.74	55.00 ± 8.64	54.77 ± 7.06	p = 0.032	p = 0.940	p = 0.024
Gender (male/female)	11/7	12/2	7/6	p = 0.235	p = 0.103	p = 0.727
BMI	23.15 ± 3.01	22.21 ± 3.01	23.62 ± 3.19	p = 0.388	p = 0.248	p = 0.677
AST (U/L)	54.46 ± 45.25	110.05 ± 100.31	109.66 ± 92.28	p = 0.044	p = 0.992	p = 0.035
ALT (U/L)	87.68 ± 88.70	137.44 ± 111.08	144.97 ± 134.27	p = −.169	p = 0.875	p = 0.163
PLT (×10^9^/L)	218.22 ± 98.68	140.64 ± 46.25	123.07 ± 49.34	p = 0.011	p = 0.349	p = 0.003
MAL/DSA	11.02 ± 1.44	8.82 ± 2.22	7.41 ± 2.03	p = 0.002	p = 0.100	p = 2.68E-06
AOL/DSA	1.94 ± 1.08	6.42 ± 4.14	12.35 ± 10.41	p = 0.001	p = 0.059	p = 0.004
Color Doppler ultrasound assessment*						
1	87.5% (14/16)	50.0% (6/12)	0.0% (0/11)	p = 0.044	p = 0.014	p = 5.98E-06
2	12.5% (2/16)	16.7% (2/12)	45.4% (5/11)	p = 1.000	p = 0.193	p = 0.084
3	0.0% (0/16)	33.3% (4/12)	36.4% (4/11)	p = 0.024	p = 1.000	p = 0.019
4	0.0% (0/16)	0.0% (0/12)	18.2% (2/11)	p = 1.000	p = 0.217	p = 0.157

*2 patients in each group were not measured by color Doppler ultrasound.

### Evaluation of LecT-Hepa, FIB-4, and APRI for estimating progression of liver fibrosis during IFN treatment of HCV-infected patients

**Figure 2:**
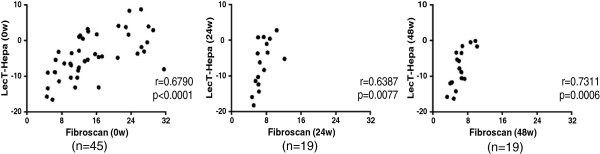
**Correlation of concentrations of LecT-Hepa and FibroScan values at baseline (0 w), 24 weeks (24 w) and 48 weeks (48 w) of the treatment process.**

**Figure 3:**
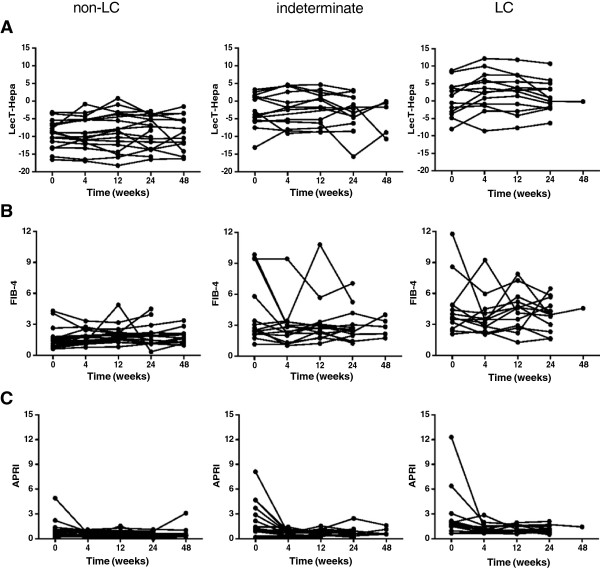
**Trend analysis of the levels of LecT-Hepa, FIB-4, and APRI during 48 weeks of IFN treatment.** Forty-five patients with CHC who achieved RVR were classified into non-LC (<12 kPa, n = 18), indeterminate (12–18 kPa, n = 14), and LC (≥18 kPa, n = 13) groups according to the degrees of severity of liver fibrosis assessed by FibroScan. Trend analysis of the levels of LecT-Hepa **(A)**, FIB-4 **(B)**, and APRI **(C)** during the treatment process in these three groups was performed.

In HCV-infected patients, evaluation of the progression of fibrosis is an important indicator of antiviral therapy [17]. However, only a few serum markers have been reported for predicting fibrosis progression and regression during treatment. Here, we investigated the relation between LecT-Hepa and fibrosis progression. First, we performed a correlation analysis of LecT-Hepa against the fibrosis levels measured by FibroScan at different times during treatment. As shown in Figure 2, we observed a significant linear correlation between the level of LecT-Hepa and FibroScan before (0 weeks R^2^ = 0.6790, p < 0.0001) and after (24 weeks, R^2^ = 0.6387, p = 0.0077 and 48 weeks, R^2^ = 0.7311, p = 0.0006) treatment. This suggests that change in LecT-Hepa reflects a change in FibroScan during IFN treatment. Then, we performed a trend analysis of LecT-Hepa, FIB-4, and APRI during 48 weeks of IFN therapy. As shown in Figure 3A, all CHC patients in the non-LC group had LecT-Hepa values <0, while the mean level of LecT-Hepa in patients with LC was >0. A gradually increasing trend of LecT-Hepa from the non-LC to the LC group was observed. The difference in LecT-Hepa between the three groups was significant at different times during treatment (p < 0.0001 for 0, 4, 12 and 24 weeks). The level of FIB-4 was also higher in the LC than in the non-LC group, and the mean level of FIB-4 in the LC group was higher than the reference cutoff value of 3.45 for cirrhosis [27]. However, this trend was not obvious and regular in all patients. In contrast, APRI showed lesser changes between the non-LC and LC groups. These results indicated that LecT-Hepa was effective for evaluation of the progression of fibrosis, at least in this cohort.

To investigate the change in LecT-Hepa during the 48-week course of IFN therapy in detail, we analyzed the levels of LecT-Hepa, FIB-4, and APRI at 0, 4, 12, 24, and 48 weeks of therapy in 45 CHC patients (Additional file 1: Figure S1 and Table 3). The mean level of LecT-Hepa was increased from −4.69 to −3.25 in the LC group (p = 0.076, paired *t* test) during the early phase of therapy (0–4 weeks), followed by a small but meaningful reduction after viral elimination (from 4 to 12, 24, and 48 weeks) (the mean value from −3.25 to −3.24, −4.19 and −7.31, p = 0.029 from 4 to 24 weeks, p = 0.026 from 12 to 24 weeks). For the other two indices, APRI showed a dramatic decrease during the early stage of IFN treatment (0–4 weeks) (p = 0.0009, paired *t* test), followed by a more stable trend (mean value from 0.81 to 0.83, 0.78 and 0.76, p = 0.275, one-way ANOVA), whereas FIB-4 showed no clear regular changes during IFN treatment. Combined with the significant correlation of LecT-Hepa and FibroScan, we suggest that the change in LecT-Hepa is superior to FIB-4 and APRI for describing the changes in fibrosis during IFN treatment in this cohort.

**Table 3:** **Levels of LecT-Hepa, FIB-4 and APRI in45 CHC patients during 48 weeks course of IFN therapy**

Weeks	0	4	12	24	48
LecT-Hepa					
Non-LC (n = 18)	−9.22 ± 3.77	−9.09 ± 4.66	−8.72 ± 5.66	−8.71 ± 4.69	−8.98 ± 4.88
Indeterminate (n = 14)	−2.10 ± 4.63	−2.14 ± 5.02	−2.12 ± 4.72	−3.47 ± 5.13	−4.40 ± 5.01
LC (n = 13)	0.83 ± 5.06	2.82 ± 5.60	2.38 ± 5.32	1.29 ± 4.39	−0.17
Total (n = 45)	−4.69 ± 6.11	−3.25 ± 7.01	−3.24 ± 6.97	−4.19 ± 6.21	−7.31 ± 5.35
FIB-4					
Non-LC (n = 18)	1.63 ± 1.06	1.72 ± 0.70	2.03 ± 0.94	1.87 ± 1.03	1.81 ± 0.68
Indeterminate (n = 14)	4.17 ± 3.11	2.79 ± 2.17	3.33 ± 2.49	2.91 ± 1.61	2.84 ± 0.91
LC (n = 13)	4.57 ± 2.71	3.80 ± 1.95	4.35 ± 1.91	3.94 ± 1.53	4.55
Total (n = 45)	3.27 ± 2.68	2.70 ± 1.85	3.15 ± 2.04	2.79 ± 1.60	2.22 ± 1.01
APRI					
Non-LC (n = 18)	0.96 ± 1.11	0.54 ± 0.27	0.58 ± 0.36	0.51 ± 0.29	0.66 ± 0.76
Indeterminate (n = 14)	2.44 ± 2.18	0.70 ± 0.32	0.76 ± 0.37	0.82 ± 0.56	0.89 ± 0.48
LC (n = 13)	2.92 ± 3.15	1.26 ± 0.61	1.19 ± 0.45	1.10 ± 0.46	1.44
Total (n = 45)	1.98 ± 2.31	0.81 ± 0.52	0.83 ± 0.46	0.78 ± 0.49	0.76 ± 0.69

### Evaluation of the role of LecT-Hepa in prognosis of patients with HCV

**Figure 4:**
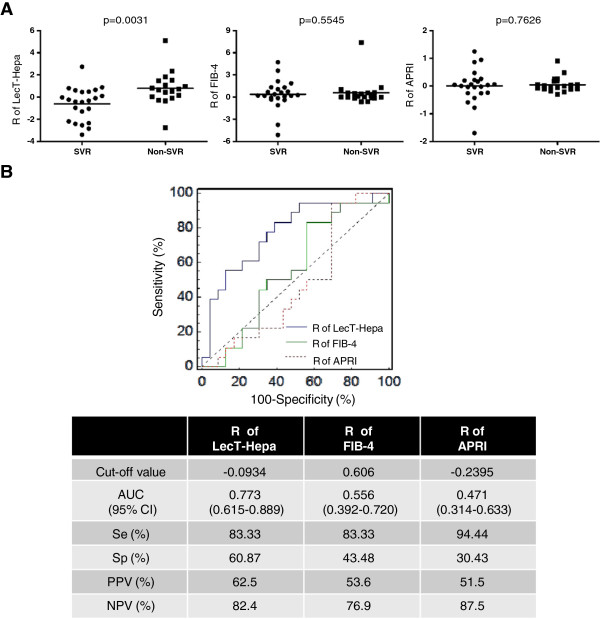
**Evaluation of roles of R-value LecT-Hepa, FIB-4, and APRI in predicting treatment outcome of HCV. (A)** Serum levels of LecT-Hepa, FIB-4, and APRI from 4 to 12 weeks (R = 12 – 4 weeks) were calculated in SVR (n = 23) and non-SVR (n = 18) patients who achieved RVR during the 48-week course of IFN therapy and underwent a 2-year period of follow-up (4 of the 45 patients were lost to follow-up). Mean values are indicated by a horizontal line and p values were calculated by the Mann–Whitney *U* test. **(B)** ROC curves of LecT-Hepa, FIB-4, and APRI for distinguishing patients with SVR from non-SVR. The cut-off values were based on the Youden’s index from the ROC curve. Se, sensitivity; Sp, specificity.

To evaluate the relationship between changes in LecT-Hepa and prognosis of CHC patients, we compared the levels of LecT-Hepa, FIB-4, and APRI in 45 patients who attained RVR with different treatment outcomes (SVR or non-SVR). Patients who attained RVR had undetectable HCV RNA after 4 weeks of therapy. We compared the clinical characteristics including the serum index between SVR and non-SVR groups (Table 4). For the serum indicators LecT-Hepa, FIB-4, and APRI, we calculated the R-value given as the sum of the changes from 4 to 12 weeks (R = 12–4 weeks) during IFN treatment, which showed the variation after viral elimination and reflected the early outcome of treatment. Besides the effect of serum HCV RNA level on treatment outcome, it is worth noting that at 12 weeks of therapy, only R value of LecT-Hepa showed a significant difference (p = 0.0031, Mann–Whitney *U* test) between SVR and non-SVR groups, while those of the other two indicators were not (p = 0.5545 for FIB-4 and p = 0.7626 for APRI) (Figure 4A). Those results suggest that the change in LecT-Hepa at the first 12 weeks of therapy was more sensitive in predicting the treatment outcome than FIB-4 and APRI were. In addition, from this preliminary result, we found that R value of LecT-Hepa were higher in patients who have not attained SVR.

**Table 4:** **Clinical characteristics of the SVR and non-SVR patients**

	SVR patients (n = 23) ^1^	Non-SVR patients (n = 18) ^1^	Significance
Age (year)	52.13 ± 7.86	53.78 ± 7.20	p = 0.4530
Gender			p = 0.7020
Male	14	12	
Female	9	6	
BMI	23.22 ± 2.98	22.27 ± 2.95	p = 0.3510
ALT (U/L)^2^	156.59 ± 134.65	80.74 ± 67.90	p = 0.0659
AST (U/L)^2^	104.59 ± 97.74	65.27 ± 59.63	p = 0.1180
PLT (×10^9^/L)^2^	138.30 ± 57.34	205.94 ± 102.76	p = 0.0551
AFP ^2^	2.99 ± 1.23	4.68 ± 2.96	p = 0.0841
HCV RNA (×10^6^ eq/mL)^2^	1.15 ± 1.60	8.78 ± 9.92	p = 0.0051
FibroScan^2^	17.16 ± 7.98	13.56 ± 7.15	p = 0.1412
Liver fibrosis assessed by FibroScan			p = 0.2170
Non-LC	7	9	
Indeterminate	6	6	
LC	10	3	
R of LecT-Hepa	−0.60 ± 1.48	0.79 ± 1.54	p = 0.0031
R of FIB4	0.38 ± 2.00	0.62 ± 1.76	p = 0.5545
R of APRI	0.01 ± 0.60	0.04 ± 0.28	p = 0.7626

^1)^4 of 45 patients were lost to follow-up.

^2)^Clinical information was the baseline (0 weeks) information.

Furthermore, to evaluate the overall diagnostic performances and attempt to establish clinically useful cut-off levels of these serum indices, we constructed receiver-operating characteristic (ROC) curves for R-values of LecT-Hepa, FIB-4, and APRI. As shown in Figure 4B, the area under the curve (AUC) (95% CI) of LecT-Hepa for distinguishing between SVR and non-SVR patients (0.773, 0.615–0.889) was superior to FIB-4 (0.556, 0.392–0.720) and APRI (0.471, 0.314–0.633), and the difference were significant between LecT-Hepa and the other two indicators (p = 0.043 *vs.* FIB-4 and p = 0.011 *vs.* APRI). Based on Youden’s index from the ROC curve, the optimal cut-off value of LecT-Hepa was −0.0934, with sensitivity of 83.33%, specificity of 60.87%, positive predictive value (PPV) of 62.5%, and negative predictive value (NPV) of 82.4%. These results implied that the change in the serum level of glycoprotein LecT-Hepa could predict the antiviral treatment response more quickly than FIB-4 and APRI, even at the first 12 weeks of therapy, which may provide more precise information for treatment protocols of CHC.

## Discussion

For patients with CHC, the traditional therapy is a combination of IFN and ribavirin. Recently, with the development of many other drugs targeting viral or host factors, and the approval of two direct-acting antiviral agents [28, 29], the question of who should be treated and with what regimen has become increasingly complex to address and needs more careful consideration [3]. Liver biopsy is considered as the gold standard for fibrosis staging. However, it cannot be used for continuously monitoring the progression of hepatitis because of its invasiveness and lack of accuracy. Thus, developing noninvasive tests like serum indictors that could continuously monitor the histological progression of hepatitis during therapy is beneficial for providing information for physicians and optimization of treatment. At present, a few biomarkers have been reported to predict the response to IFN-based regimens before the start of antiviral therapy [30–32]. For example, a recent study has suggested that patients with a favorable interleukin-28 (IL28B) genotype can receive peginterferon and ribavirin first, with the approved triple therapy subsequently if the initial treatment fails [33]. In addition, the pretreatment interferon-gamma-inducible protein-10 (IP-10) levels in plasma can predict RVR and SVR in patients infected with HCV genotype 1, and thus may be helpful in decision making regarding pharmaceutical intervention [34]. However, it should be stressed that there are few biomarkers that can monitor the progression of hepatitis during therapy. Thus, in this study, we focused on the potential predictive value of serum LecT-Hepa level during treatment with IFN and ribavirin. We analyzed the clinical information, including serum levels of LecT-Hepa, FIB4, and APRI. We clearly showed changes in serum level of LecT-Hepa during IFN treatment. We are particularly interested in the small reduction in LecT-Hepa after viral elimination (from 4 to 12 and 24 weeks) because at that time fibrosis began to ease [35, 36]. Based on the significant correlation of LecT-Hepa and FibroScan, we speculate that the change in LecT-Hepa may reflect the changes in fibrosis during IFN treatment (Figures 2 and 3). We only used RVR patients in this study; all of whom had a ≥2 log10 decrease in HCV RNA level by 4 weeks of therapy. SVR patients maintained a low or undetectable HCV RNA level during and after therapy. However, non-SVR patients showed virological breakthrough or relapse during or after therapy (Additional file 2: Figure S2). Serum levels of ALT and AST for SVR and non-SVR patients showed a similar tendency, with a dramatic decrease at 0–4 weeks, followed by a more stable trend. The HCV RNA quantitation became to decrease and the liver function returned to normal is the clinical indicators to determine the treatment outcome. During this process, LecT-Hepa showed a decrease just after viral elimination (4–12 weeks) for SVR patients while it showed a late decrease after viral elimination (12–24 weeks) for non-SVR patients (Additional file 2: Figure S2). Our data also showed that the change in LecT-Hepa was well correlated with the treatment outcome of CHC (p = 0.0031). If patients had an increased R value in LecT-Hepa (R = 12–4 weeks), they were more likely to experience relapse and become non-SVR (Figure 4).

Currently, the mechanism of relapse is not fully understood but several factors have been reported as risk factors for relapse and response [37], such as viral genotype 1 [38], high viral load [39], metabolic factors [40], shorter treatment with inadequate doses of ribavirin, and the degree of liver fibrosis and cirrhosis [41]. Previous reports have suggested that the index LecT-Hepa is one of the best candidates for glyco-indicators in liver fibrosis. LecT-Hepa count is based on the glyco-alternation in serum AGP. AGP is mainly synthesized in the liver and its glycosylation has a profound effect on collagen fibril formation [42, 43]. Goodman and Marcellin et al. have reported that the degree of liver fibrosis is characterized by a linear increase in fibrillar collagen, which was more resistant to enzymatic degradation in their studies [44, 45]. Thus, we speculate that LecT-Hepa level shows a linear correlation with the degree of fibrosis. Now, we understand the relation between LecT-Hepa level and treatment outcome. If the R value (12–4 weeks) is larger, it indicates that the degree of fibrosis at 12 weeks is more severe than at 4 weeks. That means that after treatment, liver fibrosis is not relieved and may become more severe. In other words, the treatment is not effective in these patients, and they will likely not attain an SVR. In addition, because the coagulation process did not affect glycosylation of AGP, we found that the level of LecT-Hepa showed no difference in serum and plasma. We also compared the LecT-Hepa levels in patients with HCV genotype 1a and 2b (Additional file 3: Figure S3). Those results showed that the level of LecT-Hepa was not affected by sample type or HCV genotype, and the change in LecT-Hepa level indeed reflected the therapeutic efficacy.

To the best of our knowledge, this is the first study to investigate the noninvasive serum glyco-marker as a predictive factor for prognosis of CHC patients undergoing treatment. The prognostic value of serum LecT-Hepa level is superior to that of other biochemical markers such as FIB-4 and APRI just at the first 12 weeks of therapy. In addition, because the level of LecT-Hepa is positively correlated with the degree of fibrosis, it may be used for liver function monitoring at optimal intervals and for the prediction of the treatment outcome of new antifibrotic drugs.

## Conclusions

In summary, this study was a trial for the estimation of therapeutic efficacy in patients with CHC using serum glycoproteins. It is an extension of previous study which has found LecT-Hepa as a good predictor of fibrosis using glycomics technologies. Our study revealed that the change in serum level of LecT-Hepa after viral elimination may serve as an early predictor of antiviral treatment response in CHC patients treated with IFN and ribavirin, and may provide additional information for individualizing treatment. This study provides evidence for the clinical value of serum glycomics and gives a new perspective that the serum glyco-marker could be used as a joint indicator target of disease.

## Materials and methods

### Patients

A total of 142 patients with a positive anti-HCV antibody and HCV viral load were enrolled from the Department of Hepatology, First Hospital of Jilin University. Patients were enrolled after August 2010 and followed up for at least 48 weeks. Inclusion criteria were (1): diagnosis with CHC; and (2) HCV RNA was positive as determined by the COBAS TaqMan HCV test (Roche Diagnostics, Branchburg, NJ, USA). Exclusion criteria were: (1) co-infection with another hepatitis virus or HIV; (2) excessive alcohol intake; (3) hepatocellular carcinoma or its history; and (4) decompensated liver cirrhosis.

This retrospective cohort study was divided into two parts: One part contained 97 patients with sera and plasma collected simultaneously. The other part included 213 serum specimens from the remaining 45 patients who received 48 weeks treatment with IFN and ribavirin, and were followed up for 96 weeks. All of the 45 patients achieved an RVR with ≥2 log_10_ decrease in HCV RNA level by 4 weeks of therapy. This study was in accordance with the ethical guidelines of the 1975 Declaration of Helsinki and was approved by the Ethical Committee of the First Hospital, Jilin University. Each participant gave written informed consent.

### Detection and quantification of HCV RNA

The concentration of HCV RNA in serum was determined by reverse transcriptase polymerase chain reaction using the COBAS TaqMan HCV assay (Roche Diagnostics). Serum was collected at different time points during therapy and follow-up (0, 4, 12, 24, 48, 60, 72, 96, and 144 weeks). According to the viral kinetic response and treatment outcome, 45 patients were judged as SVR with undetectable HCV RNA 24 weeks after therapy was complete, or as non-SVR.

### Clinical and biological data

The basic anthropometric parameters, such as age and sex of the patients were recorded. Serum and plasma samples were collected and stored at −80°C until analysis. The serum biochemical parameters, including concentrations of total bilirubin (TBIL), direct bilirubin (DBIL), alkaline phosphatase (ALP), γ-glutamyltransferase (GGT), ALT, aspartate aminotransferase (AST) and PLT were assessed by the medical laboratory of the First Hospital of Jilin University. The APRI and FIB-4 index were calculated according to published formulas [46, 47].

### Liver stiffness measurement

Liver stiffness was measured by transient elastography using FibroScan (EchoSens, Paris, France). The measurement depth was between 25 and 65 mm. For each patient, 10 validated measurements were performed. The success rate was calculated as the number of validated measurements divided by the total number of measurements. The results were expressed in kilopascals. The median value was considered representative of the elastic modulus of the liver. Only procedures with 10 validated measurements and a success rate of at least 60% were considered reliable.

### Automatic acquisition of quantitative glyco-alteration of AGP (LecT-Hepa)

The detailed procedure for LecT-Hepa has been described previously [22, 25]. Each individual serum or plasma sample (5 μL) was diluted and heated at 95°C for 20 min before enrichment of AGP. The AGP in the sample was enriched by immunoprecipitation with a biotinylated anti-AGP antibody using an automated protein purification system (ED-01; GP BioSciences, Tokyo, Japan). Finally, fibrosis-specific glyco-alteration of the enriched AGPs was determined by lectin-antibody sandwich immunoassays with a combination of three lectins (*Datura stramonium* agglutinin (DSA), *Maackia amurensis* leukoagglutinin (MAL), and *Aspergillus oryzae* lectin (AOL)) [23] using an automated chemiluminescence enzyme immunoassay system (HISCL-2000i; Sysmex, Kobe, Japan). The criterion formula of LecT-Hepa was as follows [22]: LecT-Hepa = log_10_[AOL/DSA] × 8.6 – [MAL/DSA].

### Statistical analysis

Statistical calculations were conducted with Microsoft Office Excel and SPSS version 16.0 statistical package (SPSS, Chicago, IL, USA). Categorical data were analyzed using χ^2^ test and continuous variables were compared with the Student’s *t* test or Mann–Whitney *U* test. In addition to assessing the predictive ability of various markers to differentiate SVR from non-SVR patients, ROC curve analysis was performed. Diagnostic accuracy was expressed as the diagnostic specificity, sensitivity, PPV, NPV, and AUC. The cutoff values were obtained from Youden’s index [48]. A p value <0.05 in all cases was considered statistically significant.

## Electronic supplementary material

Additional file 1: Figure S1: Trend analysis of the levels of LecT-Hepa, FIB-4, and APRI during 48 weeks of IFN treatment in 45 CHC patients. (PDF 25 KB)

Additional file 2: Figure S2: Clinical information for SVR and non-SVR patients at 0–48 weeks. (PDF 27 KB)

Additional file 3: Figure S3: Relation of the levels of LecT-Hepa, FIB-4, and APRI with HCV genotype. We compared the levels of LecT-Hepa, FIB-4, and APRI during 48 weeks of IFN therapy in patients with different HCV genotype (dot: HCV genotype 1b; circle: HCV genotype 2a). (PDF 23 KB)

## Abbreviations

CHC: Chronic hepatitis C
HCV: Hepatitis C virus
IFN: Interferon
SVR: Sustained virological response
RVR: Rapid virological response
ELF: Enhanced liver fibrosis
AGP: α1-acid glycoprotein
DSA: *Datura stramonium* agglutinin
MAL: *Maackia amurensis* leukoagglutinin
AOL: *Aspergillus oryzae* lectin
APRI: Aspartate aminotransferase-to-platelet ratio index
TBIL: Total bilirubin
DBIL: Direct bilirubin
ALP: Alkaline phosphatase
GGT: γ-glutamyltransferase
ALT: Alanine aminotransferase
AST: Aspartate aminotransferase
PLT: Platelet count
BMI: Body mass index
ROC: Receiver-operating characteristic
AUC: Area under the curve
PPV: Positive predictive value
NPV: Negative predictive value
IP-10: Interferon-gamma-inducible protein-10
IL28B: Interleukin-28B.
