# Supplementary figures and images for: LecT-Hepa facilitates estimating treatment outcome during interferon therapy in chronic hepatitis C patients
Source: Clin Proteomics. 2014 Dec 11;11(1):44. doi: 10.1186/1559-0275-11-44 (PMC4276098; doi:10.1186/1559-0275-11-44)

Fig.S1 Zou et al.

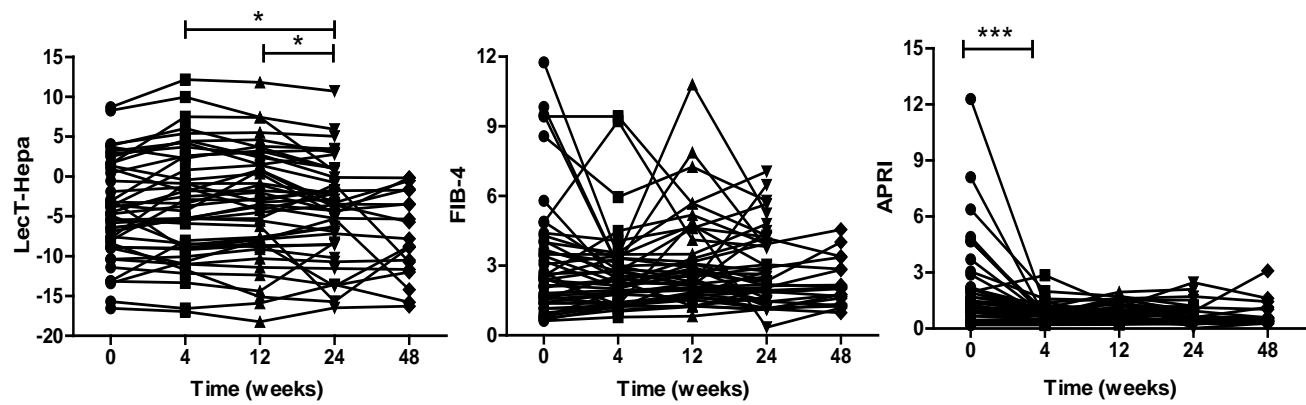

Supplement: Supplementary file 1 — Additional file 1: Figure S1: Trend analysis of the levels of LecT-Hepa, FIB-4, and APRI during 48 weeks of IFN treatment in 45 CHC patients. (PDF 25 KB) [file 12014_2014_84_MOESM1_ESM.pdf]

Fig.S2 Zou et al.

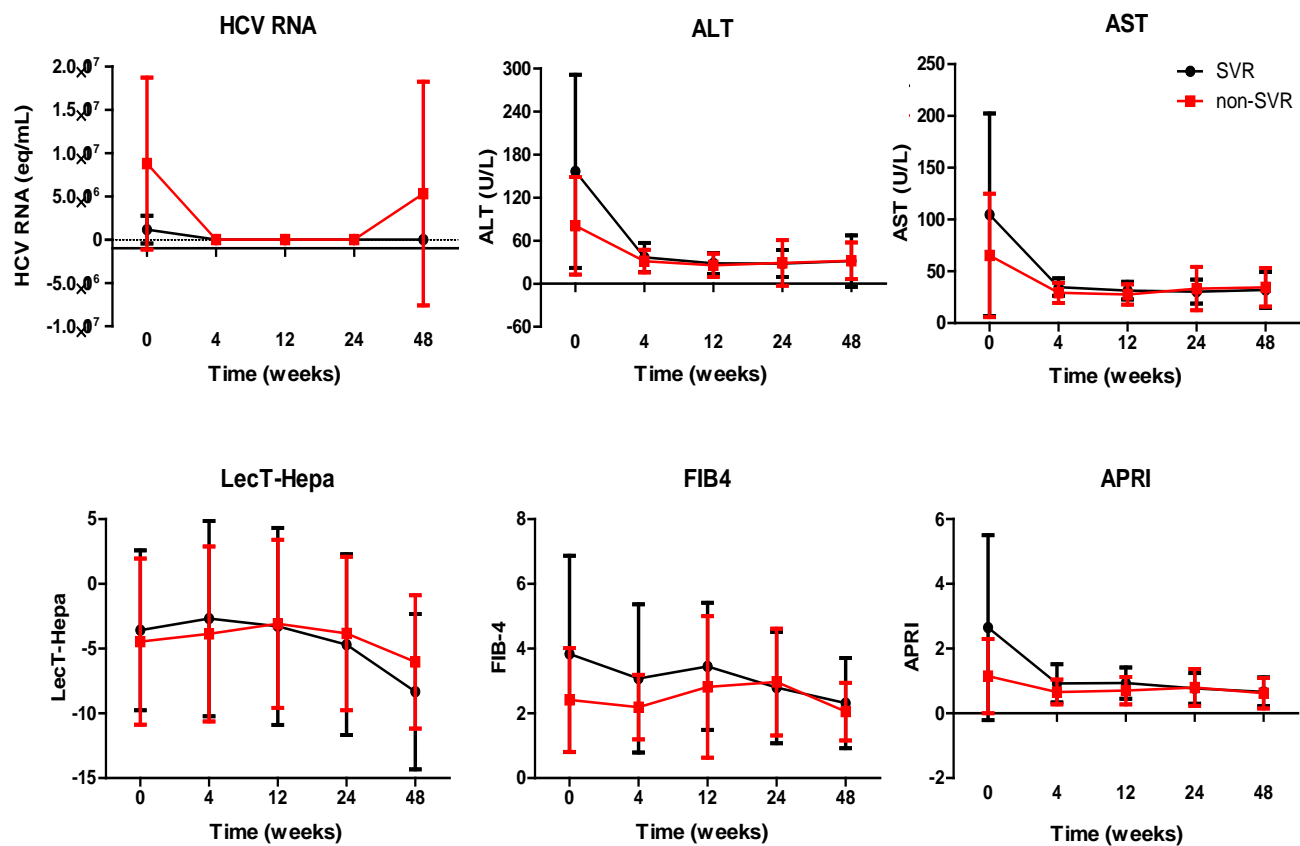

Supplement: Supplementary file 2 — Additional file 2: Figure S2: Clinical information for SVR and non-SVR patients at 0–48 weeks. (PDF 27 KB) [file 12014_2014_84_MOESM2_ESM.pdf]

Fig.S3 Zou et al.

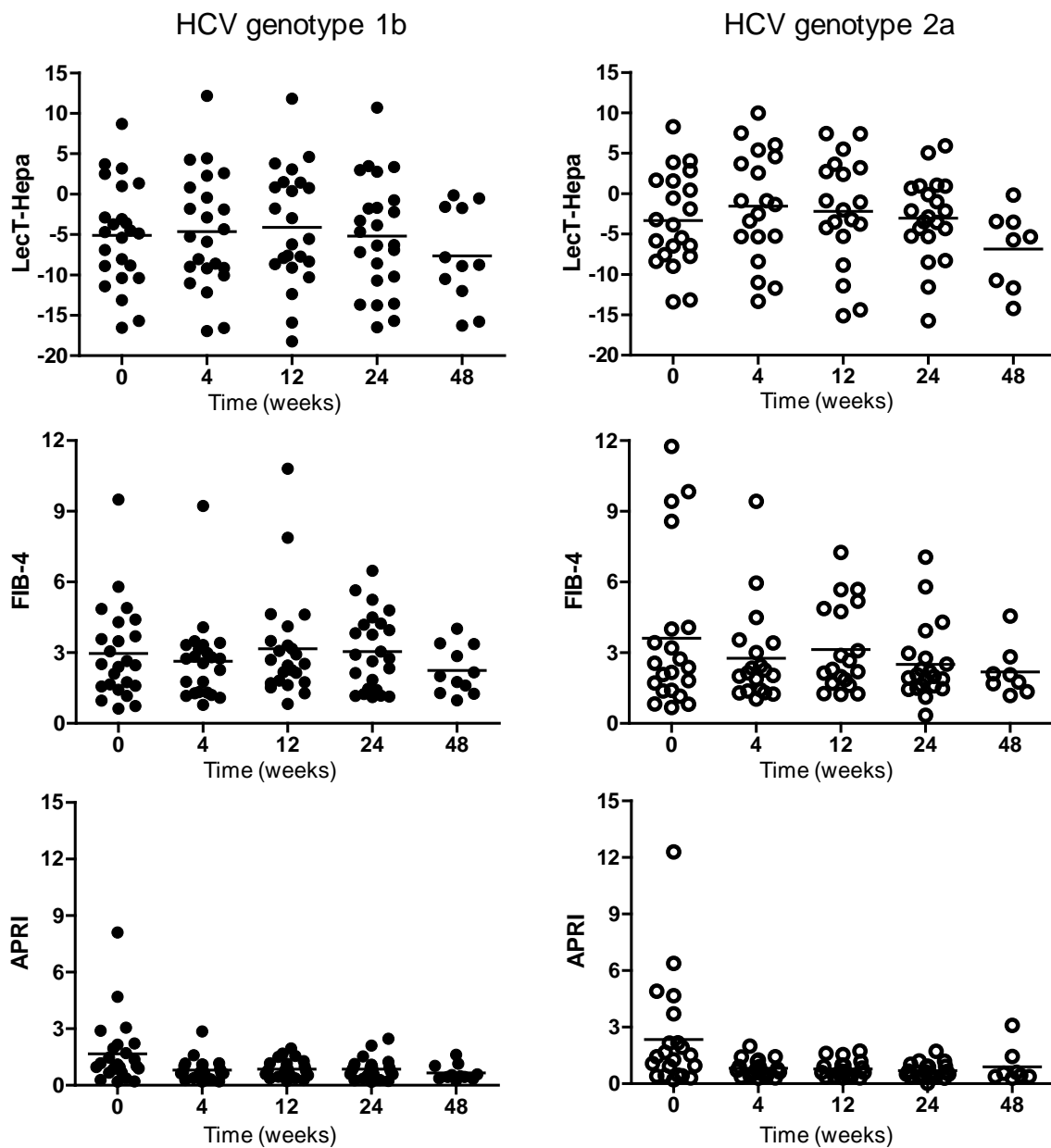

Supplement: Supplementary file 3 — Additional file 3: Figure S3: Relation of the levels of LecT-Hepa, FIB-4, and APRI with HCV genotype. We compared the levels of LecT-Hepa, FIB-4, and APRI during 48 weeks of IFN therapy in patients with different HCV genotype (dot: HCV genotype 1b; circle: HCV genotype 2a). (PDF 23 KB) [file 12014_2014_84_MOESM3_ESM.pdf]
